# *“A coalition of the willing”*: experiences of co-designing an online pain management programme (iSelf-help) for people with persistent pain

**DOI:** 10.1186/s40900-021-00275-0

**Published:** 2021-05-11

**Authors:** Hemakumar Devan, Meredith A. Perry, Mostafa Yaghoubi, Leigh Hale

**Affiliations:** 1 Centre for Health, Activity and Rehabilitation Research (CHARR), School of Physiotherapy, Wellington, New Zealand; 2 Centre for Health, Activity and Rehabilitation Research (CHARR), School of Physiotherapy, Dunedin, New Zealand

**Keywords:** Chronic pain, Co-design, Patient engagement, Participatory action, Qualitative

## Abstract

**Background:**

Participatory approaches to developing health interventions with end-users are recommended to improve uptake and use. We aimed to explore the experiences of co-designing an online-delivered pain management programme (iSelf-help) for people with persistent pain.

**Methods:**

A modified participatory action research (PAR) framework was used to co-design contents and delivery of iSelf-help. The PAR team included: (1) a patient advisory group consisting of people living with persistent pain (*n* = 8), (2) pain management service clinicians (*n* = 2), (3) health researchers (*n* = 3), (4) digital health experts (n = 2), (5) a health literacy expert, and (6) two Māori health researchers and our community partner who led the cultural appropriateness of iSelf-help for Māori (the Indigenous population of New Zealand). The iSelf-help co-design processes and activities of the ‘PAR’ team is reported in another paper. In this paper, all PAR team members were invited to share their experiences of the co-design process. Individual interviews were held with 12 PAR team members. Interview transcripts were analysed using the General Inductive Approach.

**Results:**

Five common themes were identified from the interviews: (1) Shared understanding and values of the co-design process, (2) Mismatched expectations with content creation, (3) Flexibility to share power and decision making, (4) Common thread of knowledge, and (5) Shared determination. Sustaining these themes was an overarching theme of “A coalition of the willing”.

**Conclusions:**

PAR team members valued the shared determination and responsibility to co-design iSelf-help. They also acknowledged the complexities and challenges during the process related to mismatched expectations, power sharing and establishing a common thread of knowledge. Successful co-design requires a shared commitment and responsibility as a coalition to meet the aspirations of end-users, within the boundaries of time and budget.

**Supplementary Information:**

The online version contains supplementary material available at 10.1186/s40900-021-00275-0.

## Plain English summary

Persistent non-cancer pain affects one in five people globally. Māori (the Indigenous population of New Zealand) experience a higher persistent pain prevalence than non-Māori. People living with persistent pain in New Zealand experience significant challenges in accessing specialised pain services, such as long-waiting times for referral, delays in getting a persistent pain diagnosis, lack of specialised health professionals in pain management and services that do not provide culturally responsive care. In an increasingly digital world, one way of addressing such access barriers is to provide pain services remotely. Using a participatory action research (PAR) framework, we co-designed an online version of an existing hospital-based pain management programme. The PAR ‘team’ included patients living with persistent pain, who have previously completed the pain management programme and other stakeholders including pain management clinicians, health researchers, technology design experts, and a health literacy expert. The PAR ‘team’ also included two Māori researchers, and our Māori community partner who led the cultural adaptation process of the online programme with Māori community members living with persistent pain. We invited all the PAR team members asking their experiences of co-designing an online pain management programme (iSelf-help) that is culturally adapted for Māori. Our results from interviewing 12 PAR team members suggest the shared values and determination to co-design the online programme enabled team members’ journey as a coalition to be successful, however, along the way, the process was complex and challenging at times both within and between the various teams. The key factors that caused such complexity were the mismatched expectations and understandings of the project, negotiating power sharing between the various teams and establishing a common thread of knowledge. We conclude that successful co-design requires a shared commitment and responsibility as a coalition to meet the aspirations of end-users, within the limits of time and budget.

**Supplementary Information:**

The online version contains supplementary material available at 10.1186/s40900-021-00275-0.

## Background

Patient and public engagement in the design, conduct and dissemination of health research is viewed as depoliticising health and democratisation of research process [1]. From a social justice perspective, “patients have a fundamental right to be engaged in research” [2] and there is an ethical mandate to involve patients in decision-making to enhance transparency [3]. Stemming from a social science framework, co-design approaches to collaboratively working with patients in health research and service delivery have gained momentum in the last decade [4]. Various terms are used to describe these co-design approaches including ‘co-production’, ‘co-creation’, ‘participatory research’, ‘patient engagement’, ‘patient partner involvement’, ‘integrated knowledge translation’, and ‘participatory action research’ [2, 5]. The extent of patient engagement in health services research is a continuum ranging from *consultation* (being asked about patient needs), *involvement* (being involved as patient advisors to identify solutions), and *partnership and shared leadership* (being engaged to co-design solutions and co-produce health services with healthcare providers) [6]. Despite the varied terminology and various expectations of contribution to co-design approaches, active patient engagement in all the research processes including design, delivery and implementation of health interventions is integral to minimise research waste [7], and potentially enhances the credibility, applicability and uptake of research findings [8]. Core principles guiding meaningful co-design include sharing of power, being inclusive of all voices, mutual respect to each other’s views, reciprocity, and building and sustaining relationships [9].

Despite the emerging practice toward active patient engagement or co-design approaches (henceforth referred to as ‘co-design approaches’) in health, the evidence on best practice for meaningful co-design with patients is lacking. In a comprehensive systematic review, Domecq et al. (2014) identified that co-design approaches in health research was feasible, the included studies (*n* = 142) however, did not clearly report the best practice of co-design approaches [10]. There was a perceived risk of co-design approaches becoming ‘tokenistic’ from the perspectives of both researchers and patients [10]. In a subsequent review analysing co-design approaches in published clinical trials until 2016 (including randomised controlled trials (RCTs) and controlled clinical trials), 17 RCTs reported some form of co-design in four main research processes [11]. These processes included co-designing with patients in: (1) developing the research question, (2) choosing the primary outcome, (3) intervention design, delivery and interpretation of results, and (4) dissemination of trial results [11]. None of the included RCTs in Fergusson et al. [11], review met all four processes of co-design. The included RCTs commonly adopted community-based participatory research as a co-design approach to engage patients and community partners. Almost half of the included RCTs (8/17) engaged Indigenous and culturally and linguistically diverse communities using community-based participatory approaches [11]. Overall, there appears limited number of RCTs incorporating co-design approaches to patient engagement and reporting is unclear of *how* patients were engaged and the *impact* of such engagement on patients, researchers and overall trial outcomes [2].

International research bodies acknowledge the importance of these co-design approaches leading to the development of established co-design frameworks. These include INVOLVE (supporting patient INVOLVEment in NHS, public health and social care research) from the UK [12], Strategy for Patient-Oriented Research (SPOR) from Canada [13] and the Patient-Centred Outcomes Research Institute (PCORI) from the United States [14]. There is a clear mandate for international consensus on defining meaningful co-design to engage with patients [15], need for clear reporting of co-design processes, with available checklists, such as Guidance for Reporting Involvement of Patients and the Public - GRIPP2 [16], and the need for empirical studies to evaluate the impact of co-design on health outcomes [2]. A Danish report identified shared challenges among health researchers who were unsure of the methodological processes underpinning co-design approaches [15]. This Danish report highlighted several critical questions on the purpose, best practice, and the impact of co-design involving patients in health research to understand fully the merits and demerits of co-design. In line with international literature, the New Zealand Health Research Strategy (2017–27) [17] acknowledges “strong and enduring engagement with communities and consumers” to meet the health needs of New Zealanders; however, there is no clear expectation and explanation in the strategy of *how* meaningful co-design involving patients and communities should best happen.

Meaningful co-design of health interventions is particularly relevant for people with long-term health conditions as it is essential that those who will have primary responsibility for their own management have some ‘input/control’ into the programmes that might work best for them. Co-design research involving people living with persistent pain is in its infancy. Lack of co-design approaches in persistent pain literature has been reported as a barrier to uptake of findings [18] creating a clear mandate for including people with persistent pain to inform research, practice and policy [18]. Recent initiatives from the International Association for the Study of Pain (IASP) advocate for a shift toward co-design approaches, valuing the expertise of lived experiences of people with pain to shape research and clinical priorities for improved pain management (GAPPA) [19]. Peer-reviewed scientific journals in musculoskeletal pain (e.g. *Journal of Orthopaedic* and *Sports Physical Therapy*) recently endorse the value of co-design approaches in pain research by providing a dedicated section on patients as partners in research.

Persistent non-cancer pain is a common long-term condition in New Zealand (NZ) with an annual prevalence of 19.3% [20]. The prevalence is disproportionate for Māori - the Indigenous population of NZ with an annual prevalence of 23.2% [20]. Previous experiences of racial discrimination in healthcare settings [21], lack of cultural considerations to holistic view of Māori health addressing the interconnected nature of physical, psychological, spiritual and whānau (family and significant others) aspects of health [22, 23], and a sense of stoicism and stigma with reporting chronic pain [22] were cited as possible factors contributing to disproportionate burden. Currently in NZ, persistent pain is managed by primary care services with clients requiring multidisciplinary inputs are referred to tertiary pain services. With only three tertiary pain services across NZ, long waiting times for referrals, and increasing persistent pain prevalence [24] along with significant inequities for the Māori and culturally diverse (Pasifika) populations [20, 25], accessing such services pose significant barriers for all people with persistent pain and more so for Māori population.

Digital solutions to pain management are one way of enhancing access to services [26] and may potentially improve inequities for Māori, thus we co-designed an online version of a group-based pain management programme (iSelf-help), that is culturally appropriate for Māori. We used a 5-step participatory action research (PAR) framework as our co-design approach for iSelf-help development. PAR is a recommended framework for rehabilitation intervention development, evaluation and implementation [27]. This 5-step PAR framework was originally proposed for rehabilitation intervention development and implementation; we have adapted this PAR framework for co-designing iSelf-help online intervention. Our PAR “team” included a patient advisory team of people living with persistent pain, Māori community partner and their whānau (family and significant others), health researchers including Māori researchers, pain management service clinicians, digital health experts and a health literacy expert. The co-design processes and activities of the ‘PAR’ team are reported in another paper. The co-designed iSelf-help is currently under evaluation to assess the clinical and cost-effectiveness in improving pain-related disability as compared to in-person delivered pain management programme using a non-inferiority RCT design [28]. This paper aims to gain a deeper understanding of the perceptions of our PAR team to the co-design approach we used to inform future similar studies. Specifically, the research objectives were: (1) what were the experiences of members of the PAR team to this approach to co-designing a digital health intervention; (2) what processes worked, (3) what were the challenges to the co-design process, and (4) what could be done differently in future similar co-design projects.

## Methods

This is a qualitative study underpinned by a constructivist paradigm. Based on this paradigm, we recognise that knowledge is individually or socially constructed with “emphasis placed on understanding the individual and their interpretation of the world around them (p33)” [29]. Qualitative data were collected by individual interviews as they provided opportunity for gaining deeper insights of participant experiences including sharing of sensitive information [30]. The manuscript is reported based on Consolidated Criteria for Reporting Qualitative Study (COREQ) guidelines [31] (see Additional file 1) and GRIPP2 short form checklist (see Additional file 2).

### Participants

The PAR team involved in co-designing iSelf-help comprised: (1) patient advisory group (*n* = 8) of participants with persistent pain, both males and females of different age groups, who had completed an in-person pain management programme, (2) pain management clinicians (two physiotherapists) from the local tertiary pain service, (3) three non-Māori research investigators, (4) two digital health experts from Melon Health™ (Melon Health™ is a digital company in NZ experienced in providing innovative technological solutions for people living with long-term health conditions), and (5) a health literacy review expert. The PAR team also included two Māori researchers and a Māori community partner who led the cultural adaptation process of iSelf-help for Māori living with persistent pain.

### Co-design activities

The co-design process and activities occurred over a period of nine months (September 2018 to May 2019), which were reported in a separate paper. In brief, a Nominal Group Technique was used to guide our co-design activities with the PAR team members. Five meetings were held with our PAR team members to generate ideas and derive consensus. In line with the recommendations of Māori-centred research principles [32], separate focus groups were held in a Marae (Māori meeting house) with fifteen Māori community members living with persistent pain and their whānau (family members). For this study, all PAR team members were invited to share their experiences of being involved in these co-design process and activities.

### Procedures

At onset, all PAR team members were provided information sheets explaining the processes, including that at the completion of co-design process they would be invited to an interview to explore the PAR process to date. PAR team members were invited by an independent Research Assistant (MY) to take part in individual interviews. The PAR team members expressing interest were sent an interview guide (Table 1). The interview guide was prepared by the research team (HD, LH, MY and MP) and included questions on teamwork, problem solving, relationships and organisation. It had different questions for end-users (i.e. patient advisory team and Māori community partner) and other co-design team members (e.g. clinicians and digital health experts) because of their unique roles within the PAR team (see Table 1). Interviews were semi-structured and were held either in-person (at a mutually agreeable location) or via teleconferencing software (Zoom™). All interview participants consented that the interview data will be stored and analysed by MY and other research team members HD and MP. The interviews were audio-recorded and lasted between 20 to 56 min. An oral summary was presented at the end of each interview with an opportunity to feedback. Later interviewees were asked via e-mail if they wished to add additional feedback. The in-person interviews were transcribed verbatim by a professional transcription service and Zoom interviews were transcribed using an in-built text-to-speech software, which were later verified by MY and HD.

**Table 1:** Interview guide

**Participant advisory group members + Māori whānau advisory group**	**Experiences in co-designing the online resource** Could you tell us about your role in the group? Tell us about your experiences of co-creating/designing the online resource What did you enjoy the most/least? What did you learn? **Problem solving/conflict management** Could you tell me about any issues that came up during the process and how these were dealt with? Is there anything else that you want to comment on or tell us about the process? **Organisation of meetings** Could you describe a typical meeting/hui for me and describe any specific aspects that occurred or were important to you? **Work relationships** How do you feel your contributions being valued and respected? How did you find working with the team? How happy are you with the finish product of the online resource? Were there any other ways of managing this process (e.g. procedures, rules) that you think could enable the project to run more efficiently? How did you find the level trust and expectations among the team and stakeholders who were involved in this process? Did you feel that you were the trusted person and could trust team throughout the process? PAR process At the beginning we all spoke about why we wished to be involved and what we hoped would arise from the first part of the collaborations (designing the web) how do you feel about your aspirations with respect to what we have achieved now *Can you think of any aspirations of you haven’t met?*
**Clinicians, health coach and research personnel**	**Experiences in co-designing the online resource** Could you tell us about your role in the group? **Tell us about your experiences of co-creating/designing the online resource** What did you enjoy the most/least? Any specific examples Working in a team? What did you learn? Any specific examples **Problem solving/conflict management** Could you tell me about any issues that came up during the process and how these were dealt with? Is there anything else that you want to comment on or tell us about the process? **Work relationships** How do you feel your contributions being valued and respected? How did you find working with the team? How happy are you with the finish product of the online resource? Were there any other ways of managing this process (e.g. procedures, rules) that you think could enable the project to run more efficiently? How did you find the level trust and expectations among the team and stakeholders who were involved in this process? Did you feel that you were the trusted person and could trust team throughout the process? PAR process At the beginning we all spoke about why we wished to be involved and what we hoped would arise from the first part of the collaborations (designing the web) how do you feel about your aspirations with respect to what we have achieved now *Can you think of any aspirations of you haven’t met?*

### Data analysis

The General Inductive Approach was used to analyse the interview data [33]. This analysis approach was chosen, as our research objectives were evaluative and data-driven. Based on this inductive approach, MY read all the transcripts multiple times and coded text that addressed the research objectives. Similarly, HD and MP independently coded three interviews each. These three researchers then met to discuss their coding schemes and to collapse their multiple codes into three broad categories. Following this, HD independently coded all transcripts with these three categories; LH independently coded three interviews as a verification process. Two subsequent consensus meetings were held between LH, MP and HD to finalise the coding and to further collapse the broad categories into themes and subthemes. Mind maps were used in these meetings to makes sense of the coded data and iteratively derive the final themes. NVivo® (Version 12, QSR International) was used to organise the coded data.

### Trustworthiness of analysis

A summary of the results was circulated to the interviewed PAR team members to ensure the analysis represented their views and their additional insights were sought. The interviews conducted by an independent research assistant (MY) who was not involved in the co-design process ensured that PAR team members were able to freely share their experiences. The iterative nature of analysis and involvement of multiple team members to derive consensus enhanced credibility of the analysis. Key decisions made during data collection and analysis were logged in an audit trail to ensure trustworthiness of the analysis process.

#### Reflexivity

The independent research assistant (MY) had no prior relationships with interviewed PAR team members other than the two research investigators (HD and MP). HD, MP and LH have physiotherapy backgrounds with a combined experience of greater than 50 years and MY’s a physical education background. All have experience in qualitative research with HD, LH and MP’s combined qualitative expertise is greater than 35 years. All researchers who led the analysis of this study are non-Māori belonging to different ethnicities (Indian, Middle Eastern and New Zealand European).

## Results

Twelve PAR team members took part in the individual interviews of which three were from the patient advisory group, two clinicians, two technology design members, two researchers, a Māori community partner and a Māori researcher, and a health literacy expert (Table 2). Six out of twelve interviewees responded to member check invitation and agreed with our summary findings requesting no further changes.

**Table 2:** Interviewed PAR team members (*N* = 12)

PAR team roles	Acronyms
Lived experience advisory group	PAG 1
Lived experience advisory group	PAG 2
Lived experience advisory group	PAG 3
Pain clinicians	PC 1
Pain clinicians	PC 2
Technology expert – Melon Health	TE 1
Technology support co-ordinator – Melon Health	TEC 2
Māori community partner	MCP
Māori lead researcher	MCR
Health literacy expert	HLE
Health researcher	R1
Health researcher	R2

*PAR* Participatory action research, *PAG* Patient advisory group member, *PC* Pain clinicians, *TEC* Tech expert co-ordinator, *MCP* Māori community partner *HLE* Health literacy expert, *R* Research personnel

**Fig. 1:**
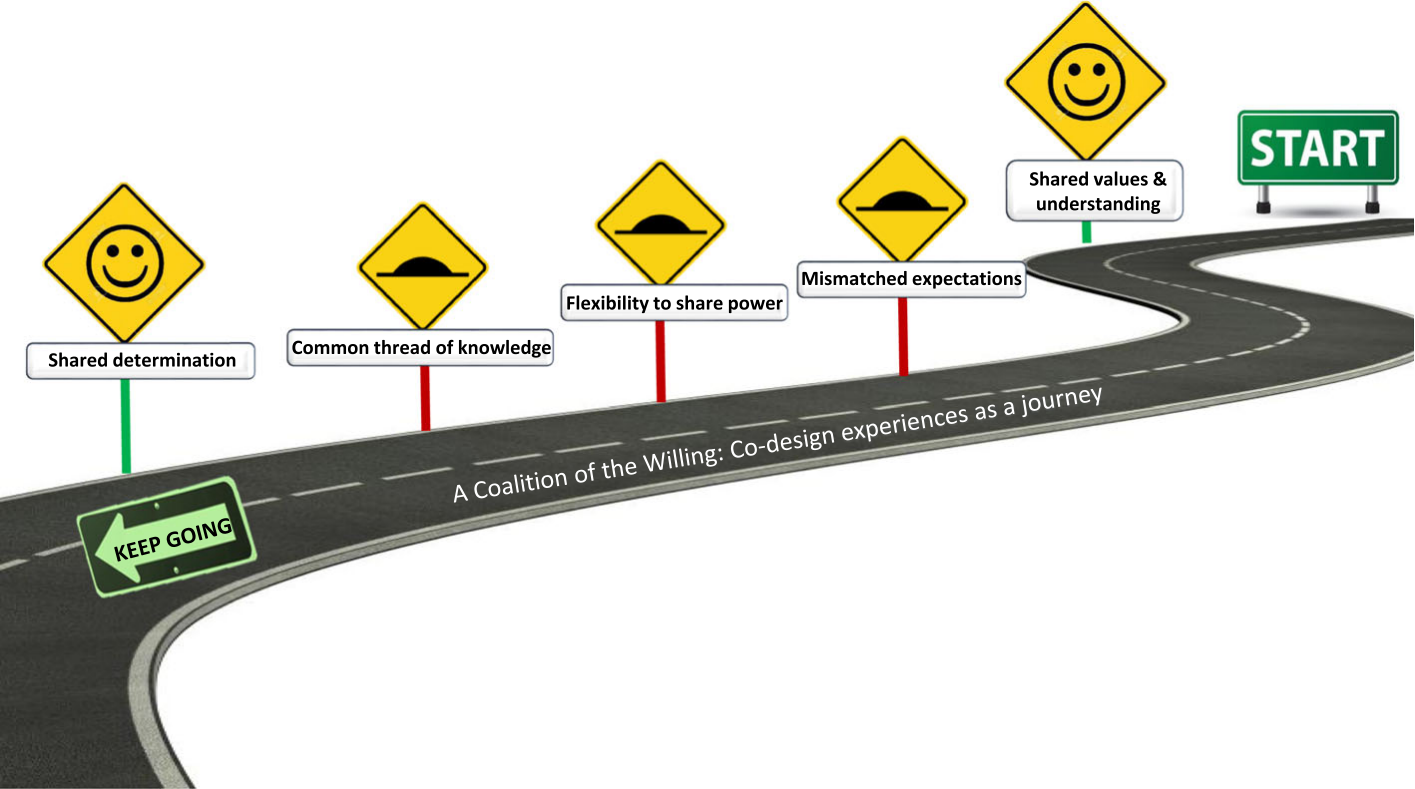
Co-design experiences illustrated as a journey of collective goodwill and tenacity despite complexities along the way

The overarching theme - *a Coalition of the Willing* – depicts a collective goodwill and shared determination of PAR team members to co-design iSelf-help despite the journey being complex, as illustrated below in Fig. 1. Five themes describe these complexities: (1) *Shared understanding and values of the co-creation process*, (2) *Mismatched expectations with content creation*, (3) *Flexibility to share power and decision makin*g, (4) *Common thread of knowledge*, and (5) *Shared determination*. Below we describe each theme, highlighting the shared experiences and complexities of the co-design process and end with how these five themes were linked by an underpinned overarching theme of - *a Coalition of the Willing.*

### Shared understanding and values of the co-design process

The PAR team members valued the experiences and the shared responsibility to co-designing an online version of a pain management programme. The processes gave an opportunity to bring the voices of a diverse range of stakeholders with varying expertise, all working together iteratively to co-design an online programme that meet the aspirations of people living with persistent pain and their whānau (family and significant others).

*“I felt that everyone had a chance to be heard, and when we got to see what they [the technology design team] produced from the information we gave them was impressive … They heard exactly what we have said and had produced some content that … was able to deliver those messages that … we’re trying to teach through programme.” (Patient advisory group member).*

The iterative nature of the meetings, particularly the face-to-face meetings, helped to facilitate a shared understanding, fostered developing meaningful relationships and establish mutual trust amongst various stakeholders. Particularly, the patient advisory group and Māori community members valued the opportunity to provide iterative feedback and were pleased when the final programme reflected their co-design ideas.

*“We were able to incorporate a lot of the recommendations as part of the landing page which was great. It was really inclusive process all the way through” (Māori community partner).*

Values derived from the co-design process varied amongst stakeholders. From the perspectives of the patient advisory group members, an opportunity to help those in similar circumstances and the feeling of being heard were key drivers for their contribution to the process. Clinicians valued the opportunity to review, reflect and revise clinical contents from the perspectives of patient advisory group. For Māori community members, as none had previously attended an in-person pain management programme, they felt unable to comment on the content of iSelf-help but were particularly happy about that cultural appropriateness of the welcoming page to the website which enabled them to *“feel that they are welcomed” (Māori community partner).*

(Note: The welcoming page (https://www.melonhealth.com/programs/pain/) consisted of a short video of Māori community members welcoming to the pain programme along with karakia (prayer) in audio-visual format, native imagery, and whakatauākī (Māori proverb). The use of simple language with less jargon using both Te Reo (Māori language) and English was included.).

### Mismatched expectations with content creation

Despite recognising the valuable experiences that arose from the co-design process, there were various understandings of the term ‘co-design’. The interpretation of this term varied between individuals of one stakeholder group and between different stakeholders, and this led to mismatched expectations between and within teams. The fluid nature of the co-design process and working within time and budget constraints, compounded miscommunication and frustrations among various stakeholders.

*“I would say for co-design overall, I think it will be important to get that shared understanding of where the project is going and what the intended outcomes are … I understand that changes over time however, given that we were on different definitions, [differences] could have been resolved a lot earlier if it had just been like “well, this is my definition of how we go about it”. That would have saved some time about who we were co-designing with and … our audiences and who was involved, who was consulted, who was actually helping to co-design. [That understanding] was different between the different partners I think.” (Technology design team).*

For example, PAR team members shared the inherent challenges of generating new content and formats of delivering content online (e.g. videos, animations and illustrated texts) based on what the patient advisory group members wanted and adapting existing contents from in-person delivered programme to an online medium.

*“The way we had to create the online stuff ended up being not particularly a good flow for us … It would have been easier for us to say: let’s have a look at what we want to have in the module and then create a video that covers it and talks about the session but that was not how that was working in XX. XX needed to do the videos first and all the written parts at the end, which was based on their timeframe and plan … So that was challenging.” (Pain clinician).*

As the module objectives and content of the in-person PMP had not been formally created into written session plans previously, it was challenging to then culturally adapt the content to meet the aspiration of Māori community members. This in turn influenced the extent and quality of cultural adaptation of iSelf-help.

*“I think the fact that this programme was not written up was the most difficult thing probably for everyone … We had to wait for the content to be written up for the online programme (iSelf-help) to overlay and then put a bit of the cultural concepts to it. It was not an ideal way and it was difficult.” (Māori lead researcher).*

As there were no set rules for finalising the co-design contents and due to the iterative nature of co-design process, the scope of work required for creating contents increased over time, which led to frustration and conflicts with the process by various stakeholders.

“*Well over 140 resources were made with a lot of XX resource in terms of text, [what] was given to us was not really in a format that was suitable for online use … So really things just got so stressed and under pressure and so with the clinicians repeating themselves three times and then us re-doing work. We were not very efficient as a team. I’d say that there would be a number of documents that would have been worked on four or five times by four or five different people. So that was part of that scope creep that nobody really realized how much there was …*” (*Technology design team).*

### Flexibility to share power or decision making

Stakeholders shared their reflections of critical incidents of conflict and miscommunication between and within teams because of mismatched expectations of co-design. These incidents demonstrated the challenge of power sharing between teams in a participatory framework with ‘*trying to satisfy everyone’s needs*’ while making key decisions.

*“Worst task was probably just the challenge of trying to keep everyone happy. We here at XX we were the people that were hosting it. And so we had to keep YY happy, but we also had to make sure that the people that were initiating the programme and actually being the face of the programme were happy with the content that we’re putting out, so that was a very hard line to walk in terms of budget and time as well.” (Technology design team).*

The researchers attempted to facilitate liaison across the various teams, negotiating across critical incidents however, these were not always resolved to all members’ satisfaction. For example, the PAR team member below described feelings of disappointment from not coming to a resolution on the tone and look of one of the online elements.

*“I guess I stopped providing some input to this because it did not seem that it is going to be received well. So I let them do their things and not having any confrontations and it did not seem that they wanted to come and ask more, which was disappointing.” (Māori lead researcher).*

Ultimately, issues were resolved by focusing on seeking feedback from ‘*who matters the most’*, namely the patient advisory group and the Māori community members with pain.

*“I got a very upset phone call from XX who felt that what had been put up would never meet the needs of the participants and it really wasn’t up to the participatory action process. So this was a very long conversation where I … I really respected their knowledge of the evidence around Web content, because that is not my area. However, perhaps the best idea would be to allow the participants to give us their feedback. So, when we had our feedback sessions with the participants, I very pointedly asked, ‘How do you find the number of resources? Are there any we should get rid of? Are any of them too academic?’ Almost all of them said, ‘Well, that is fine because there’ll be some people that want really high level stuff’.” (Health researcher).*

### Common thread of knowledge

Knowledge sharing in terms of key decisions made during the co-design process was influenced by workforce flow (i.e. people leaving teams and new people with limited experience or project background joining the team). For some, lack of involvement since the inception of co-creation process was perceived as a barrier to active participation and in some instances led to miscommunication. For some, inexperience influenced the quality of co-designed contents.

*“I got involved quite a lot when the project was quite a way down the track. And so, it changes how you work because if I was doing this from the beginning, I would have been trying to help with the framing and trying to help with the curation of the topics and how they build on each other. But when you come in, like, you basically go to say, what do you think of the problems and how can I help? And resolve the issues that have been identified*.” *(Health literacy expert).*

A few stakeholders acknowledged the need for an overall project manager to ensure a common thread of knowledge is maintained throughout the co-design process and particularly when working close to deadlines.

*“Especially, when we think back and what I said before about not having someone at top knowing that what is going on across a whole variety of teams. I think if we had that, communication would have been better.” (Technology design team member).*

Technological project management software that met all the different stakeholder standards was also perceived as a barrier for iterative feedback and establishing a common thread of knowledge.

*“So there was a kind of a mismatch between what XXX work with, and for them to be able to access the [University’s software] and vice versa … and clinicians were nowhere in between, like, they couldn’t get access to either. So I think that was one of the common reasons for the miscommunication to happen … I realized how important it is to develop those project management skills which kind of goes back to the common platform or data storage or cloud platform where people can see what everyone’s doing and that would have relieved a lot of anxiety.” (Health researcher).*

### Shared determination

Stakeholders reflected shared determination and grit amongst PAR team members to get the co-design process work as key attributes to navigate the challenges during the co-design process.

*“This process (co-design) really created a team dynamic that I had never been a part of before. You have almost put your thoughts, sweat and tears literally into creating this (online programme), everyone was passionate, and that created a beautiful and interesting bond within a team. Therefore, that was an amazing thing to experience … when we all got towards the end, and had a morning tea. It was just nice to be in the room with people having a finished product and not talk about it, we just talked about different things and it was an amazing experience to be part of it really” (Tech design team member).*

For Māori community members, the cultural sensitivity exhibited by the researchers during the co-design process was perceived to contribute to developing meaningful relationships with the community, which was valued as an important aspect for success.

*“This was the first time I have worked alongside XX (the researcher) and we have developed a really awesome relationship along the way and I always felt like XX was really mindful and respectful of Tikanga Māori (Māori way of doing research) so he would always make ensure at all stages we were mindful of including any Te reo. He was more than happy to have that critique and make sure that it was to apply those. Just the way he worked with the whānau (Māori community members) as well and everyone who have worked with him. I think that was an important part of the process.” (Māori Community partner).*

### Overarching theme: a coalition of the willing

This overarching theme sustained the success of this project. Despite the mismatched expectations and communication challenges, all PAR team members were proud of the final co-designed online programme that met many of the aspirations of all stakeholders. Particularly, everyone reflected having a co-desire to navigate this process with give and take along the way (Fig. 1).

*“Overall, I feel quite proud about what was achieved and how many voices it brought together. So yeah, it is now that we are on the other side of it. I am quite pleased with it. (Tech design team member)”.*

To have cultural aspects of pain management included in the online modules and a dedicated welcoming webpage to the programme was quite satisfying.

*“I think we have achieved what we wanted to achieve with regard to Māori content. I feel very happy [with] what has gone to that page/website. I can see the recommendations from whānau and focus groups and our thoughts initially on what would be really good to see on that page for the pain management website and clinic. What we see in there is cultures coming together and it has been done very tastefully. In addition, the feedback we have received from the whānau (Māori community members) was positive and they like the welcoming page.” (Community liaison).*

Patient advisory group members acknowledged the co-designed online programme would be a very useful resource for similar others living with ongoing pain. They also provided suggestions for further refining and re-organisation of information in online modules.

*“I think it looks fantastic. I think it will be a really valuable resource. I gave some feedback when we were looking at the prototype, there were some stuff that I thought that could be arranged differently or organised differently in terms of content. But the actual content looks really good and I think it will be a super useful product to people who need that service for patients with chronic pain.” (Patient advisory group member).*

## Discussion

This study explored the experiences of a co-design process of various PAR team members involved in developing an online-delivered, group-based pain management programme (iSelf-help); an online programme developed based on an existing in-person programme. As illustrated in Fig. 1, overall, the shared values and determination to co-design the online programme enabled PAR team members’ journey as a coalition to be successful, however, along the way; the process was complex and challenging at times both within and between the various teams. The key factors that caused such complexity were the mismatched expectations and understandings of the project, negotiating power sharing between the various teams and establishing a common thread of knowledge. The complexities were compounded by the project’s constraints of time and funding. We discuss below what processes worked as a team and what the challenges were with implications for future co-design studies.

### What processes worked?

Shared determination from all the stakeholders was perceived as key driver to successful completion of the co-design process. All stakeholders valued the underpinning philosophy and opportunity to co-design an online pain management programme by bringing the voices of different stakeholders together. The co-design process particularly provided patient advisory group and Māori community members with pain an opportunity to share their insights from a lived experience perspective, and this both validated the final product as well as providing these groups a sense of validation of their knowledge and understandings [34]. As this was this groups’ first experience of being involved in a co-design process, it provided an opportunity to share their knowledge gained from their lived experience perspective with others in similar circumstances and to also build their own knowledge by going through some of the contents of the programme provided by others. Although there are no similar studies in people with persistent pain, feelings of increased self-esteem by contributing and feeling empowered were reported in previous patient engagement studies conducted in other long-term health conditions [35]. The patient advisory group and Māori community members valued the iterative nature of the meetings, as although perceived as time-consuming and logistically challenging, they helped to develop trust and relationships over the project period of eight months. Overall, these findings align with NIHR INVOLVE’s core co-production principles of valuing each other’s views, reciprocity and building and maintaining relationships [12].

Shared relationships, cultural sensitivity and establishment of mutual trust were reported to develop meaningful engagement for Māori community members. This process of establishing meaningful relationships ‘*Whakawhanaungatanga*’ is one of the key principles of Kaupapa Māori Research framework (an Indigenous framework for Māori research) [32]. Cultural considerations to meaningful Māori engagement included, all meetings were led by our senior Māori researchers and the Māori community partner. The meetings were held face-to-face and in a Marae (Māori meeting place), which enabled Māori community members to feel comfortable to share their pain experiences and insights on the online programme. The iterative feedback to co-create the welcoming page (https://www.melonhealth.com/programs/pain/) to the programme and inclusion of whānau stories of pain in the online modules was suggested as a way of helping others to learn and care for each other. Further, at follow-up meetings, Māori community members were presented a summary of the previous meeting discussions and decisions, the transparency of this strategy further enhanced the trust of the community with the researchers. This strategy was adopted as historical mistrust and lack of transparency in decision making, cultural insensitivity and not reporting to the community following taking part in research has been reported to negatively influence trust and relationships for Māori community members [36] and other Indigenous populations globally [37].

### What were the challenges?

Despite the shared determination and mutual trust of all stakeholders involved, managing expectations around different interpretations of ‘co-design’ was a challenge. Variations in interpretations of ‘co-design’ contributed to miscommunication and frustrations *during* the co-creation process for some stakeholders, a finding similar to that reported in previous co-design studies [38]. Although the researchers pre-empted this challenge by explaining the meaning and context of co-design process to the various stakeholders at the agenda setting meeting and by having regular face-to-face meetings with various stakeholders, the interpretations of co-design process and expectations continued to be varied between and within team members, leading to miscommunication and misunderstanding of their roles. For example, a few stakeholders reported lack of written content for the in-person programme as a common challenge. However, the co-design process initially started with the patient advisory group members, and they stated their preferred type or format of resources (e.g. videos, animations and interactive texts) for each online module. Only after this step could the clinicians write the contents for each online module in the preferred format, causing a delay. This delay then meant that the Māori community members and Māori researchers could not comment timeously on the appropriateness of contents from a Māori worldview. While pain clinicians were receptive to co-develop culturally sensitive resources (e.g. welcoming page, use of metaphors and imagery, patient stories) as informed by Māori community members, it also required clinician upskilling in delivering those resources as part of the programme. Limited clinical capacity and time constraints meant clinicians were unable to sufficiently invest time to confidently deliver those resources, which also led to mismatched expectations during the co-design process.

Further, Māori community members unlike the patient advisory group members had never attended the tertiary pain service, limiting their understanding of what such a programme is like. While we consulted our Māori community partner and Māori researchers prior to our project proposal, deeper engagement with Māori community members *during* the co-design process highlighted structural discrimination, related to lack of acknowledgement of chronic pain experiences at primary care level, limited referrals to specialised pain services, and physical access barriers for Māori patients to specialist services [25]. The health inequities perpetuated from these sorts of biases within our current health systems have started to be acknowledged only relatively recently in New Zealand but relate back to colonisation. While addressing these structural factors are beyond the scope of this project, the engagement with local Māori has led to other collaborative community-led projects [39] which hope to challenge structural bias and deliver community developed, culturally appropriate pain services based on Māori worldview [39].

Another challenge was the iterative nature of co-design process, which meant there was a lack of predefined rules, resulting in the scope of the project increasing more than anticipated. For example, the number and type of co-designed resources increased, which increased the time of content creation and reduced time for feedback leading to further stress among various stakeholders. While the patient advisory group members informed the type and number of resources, clinicians and researchers wrote most of the contents. When the patient advisory group members then reviewed and approved the final contents, discussions specifically by researchers centred on whether this was ‘*true co-design*’ due to limited involvement of patient partners *during* content writing and the creation process. Such ethical dilemmas were previously reported by health researchers as “adequate integration of patients and their perspectives into research as very difficult to achieve, due either to lack of resources and experience, or to the nature of the research conducted” (p7) [40].

As the researchers led the funding application process, it was difficult to pre-empt the extent and nature of co-design process. This had an impact on accurately estimating the funding and timeline of the co-design process. The balancing act of wanting to meet the needs of the patient advisory and Māori community members within the constraints of an already fixed budget demanded limiting the number of personnel and the extent of iterative feedback from patient advisory group and community members. As personnel changed, there were new members who joined late in the co-design process, and this further influenced the common understanding and knowledge between stakeholders. This was particularly the case for the Health literacy expert who was asked to review contents of the online programme. Informed by our Māori community engagement, we made the decision to include health literacy expert *during* the co-design process, not upfront at the beginning. This decision added to another layer to the iterative content creation process, however, the inclusion of health literacy expert as part of PAR team further enriched the intervention development. Our findings concur with a realist synthesis on patient engagement processes, which concluded for meaningful patient engagement to occur, both *context* (e.g. adequate funding, time and training) and *processes* (e.g. clearer planning of how and whom to involve and individual roles of stakeholders) will influence the attitudes toward patient engagement and thus impact the *outcomes* from patient engagement [35].

### What are the implications for future co-design studies?

Our findings show that developing shared understandings of stakeholder group identities and roles is crucial to the co-design process and this takes time, but time that is well spent, as we have demonstrated meaningful engagement with Māori community members. Future studies should not under-estimate the time and funding required to enable a robust and efficient co-design process. Specifically, ensuring there is appropriate compensation for patient or community partners is in line with previous patient engagement studies [35]. Guidelines [41, 42] on *how* to discuss compensation requirements with patient advisory group members are useful resources for prospective co-design researchers. Having risk mitigation strategies in place and training for researchers on conflict resolution during co-design process would be helpful when problems arise within and between stakeholder groups. To oversee the co-design processes and provide suggestions for co-design teams involving various stakeholders, we recommend having an external co-design reference group similar to a data monitoring committee for a RCT. From a New Zealand perspective, we acknowledge international guidelines from INVOLVE [12] and CIHR [43] on *‘how’* to plan and conduct co-design studies as a useful guide. As patient engagement is an evolving method, we recommend a strategic leadership initiative by the Health Research Council (HRC) of New Zealand to adapt such guidelines to reflect the New Zealand context and to fund appropriately to ensure patient expertise is acknowledged to meet the goals of New Zealand Health Research Strategic Priority (2017–27) [44]. Currently, guidelines on developing and maintaining meaningful partnerships with Māori and Pasifika communities are lacking and there is a need for professional development opportunities for health researchers involved in patient engagement research.

Finally, to minimise miscommunication, although there was an initial agenda-setting meeting and bespoke meetings with individual teams/stakeholders, having regular meetings and a common data sharing software with all stakeholders in the PAR team would have ensured a common thread of knowledge even with new people joining the teams. While such updates occurred via e-mail, dedicated times with the PAR team to ensure shared understanding of all the stakeholders will enhance communication and address any mismatched expectations. Further, as the co-design process was conducted pre COVID-19, while face-to-face meetings were preferred, due to work and travel commitments, having the option of videoconferencing could have maximised the number of attendees for the PAR group meetings. Future studies could offer both in-person and online meetings to facilitate co-design process and evaluate the impact on developing mutual trust and relationships over time.

### Strengths and limitations

This paper reports the first online pain management programme to be co-designed with people living with persistent pain and culturally adapted to Māori community members. Another strength of this project was that the researcher (MY) who was not involved in the co-design process independently conducted the interviews and initial analysis and our senior investigator (LH) who was only involved at the macro level during the co-design process verified the analysis findings. Whilst using this co-design approach can be considered a strength of our approach, we acknowledge the following limitations that may have led to the misunderstandings and tensions reported in our findings. First, the project duration and base contents was determined based of an already pre-existing in-person delivered pain management programme. However, on discussions with patient advisory group members, the core contents of the in-person programme and some structural elements (exercises and moderated group discussions) were retained to ensure dose comparability within the non-inferiority RCT. Further, iSelf-help was developed as an alternative method delivery of an existing in-person pain management programme, delivered by the clinicians. Thus whilst the mode of content delivery could change, the core principles of the content could not. Despite this, the co-design process has led to co-creation of new contents in the form of short videos, interactive texts, animations, people’s peer-support stories and relaxation podcasts. The Māori engagement resulted in a co-created welcome page to the programme, including visuals, imagery and stories of similar others sharing their pain management experiences. We again acknowledge the structural inequalities this project has highlighted for Māori and we continue to work constructively with the Māori community to mitigate these barriers. Another key outcome from the co-design process was the inclusion of peer-support facilitator as part of iSelf-help delivery. One participant from our patient advisory group member has been invited to be our peer-support facilitator, and is currently involved in iSelf-help delivery.

Next, while some the patient advisory group members verified the results of this study, considering the ICMJE authorship criteria, we were unable to include them as patient co-authors in this particular manuscript and thus acknowledged their valuable insights and contributions in the ‘acknowledgement’ section [45]. We are in the process of submitting another manuscript explaining the co-design process of iSelf-help, which will include our patient advisory group as co-authors because they were involved in the intervention design and development and feedback on the overall manuscript. Lastly, we endeavoured to involve our patient advisory and Māori community teams in all phases of the research project, however, due to the number and type of resources that our patient partners wished to be in the online programme (*n* = 140), the extent of patient involvement *during* content creation process was limited. Despite this, our study adds to the knowledge base of co-designing an online pain management programme using a ‘team’ based co-design approach including clinicians, cross-disciplinary professionals, patient partners and Māori community members.

## Conclusions

The shared values and determination to co-design the online programme enabled PAR team members’ journey as a coalition to be successful, however, along the way; the process was complex and challenging at times both within and between the various teams. The key factors that caused complexity were the mismatched expectations and understandings of the project, negotiating power sharing between the various teams and establishing a common thread of knowledge. Developing shared understandings of stakeholder group identities and roles and shared determination as a coalition are crucial attributes to foster meaningful engagement *throughout* the co-design process to meet the aspirations of end-users, within the boundaries of time and budget.

## Supplementary Information

**Additional file 1.**

**Additional file 2.**

## Data Availability

The data from the interviews analysed during the current study are available from the corresponding author on request.

## Abbreviations

COREQ: Consolidated Criteria for Reporting Qualitative Study
PAR: Participatory action research
PCORI: Patient-Centred Outcomes Research Institute
RCT: Randomised controlled trial
SPOR: Strategy for Patient-Oriented Research
