# Supplementary material for: “A coalition of the willing”: experiences of co-designing an online pain management programme (iSelf-help) for people with persistent pain
Source: Res Involv Engagem. 2021 May 11;7:28. doi: 10.1186/s40900-021-00275-0 (PMC8112221; doi:10.1186/s40900-021-00275-0)
Supplement: Supplementary file 2 — Additional file 2. [file 40900_2021_275_MOESM2_ESM.docx]

| **Section and topic** | **Item** | **Reported on page No** |
| --- | --- | --- |
| 1: Aim | Report the aim of PPI in the study | 7 |
| 2: Methods | Provide a clear description of the methods used for PPI in the study | 6-9 |
| 3: Study results | Outcomes—Report the results of PPI in the study, including both positive and negative outcomes | 11-18 |
| 4: Discussion and conclusions | Outcomes—Comment on the extent to which PPI influenced the study overall. Describe positive and negative effects | 18-26 |
| 5: Reflections/critical perspective | Comment critically on the study, reflecting on the things that went well and those that did not, so others can learn from this experience | 19-24 |

**Additional file 2: GRIPP2 short form**
